# Mesenchymal Stem Cell-Derived Extracellular Vesicles: Seeking into Cell-Free Therapies for Bone-Affected Lysosomal Storage Disorders

**DOI:** 10.3390/ijms26136448

**Published:** 2025-07-04

**Authors:** Andrés Felipe Leal, Harry Pachajoa, Shunji Tomatsu

**Affiliations:** 1 Centro de Investigaciones en Anomalías Congénitas y Enfermedades Raras, Universidad Icesi, Cali 760031, Colombia; lealb.af@javeriana.edu.co (A.F.L.); hmpachajoa@icesi.edu.co (H.P.); 2 Centro de Investigaciones Clínicas, Fundación Valle de Lili, Cali 760001, Colombia; 3 Institute for the Study of Inborn Errors of Metabolism, Faculty of Science, Pontificia Universidad Javeriana, Bogotá 110231, Colombia; 4 Nemours Children’s Health, Wilmington, DE 19803, USA; 5 Genetics Service, Fundación Valle de Lili, Cali 760001, Colombia; 6 Faculty of Arts and Sciences, University of Delaware, Newark, DE 19716, USA; 7 Department of Pediatrics, Thomas Jefferson University, Philadelphia, PA 19107, USA

**Keywords:** extracellular vesicles, lysosomal storage disorders, mesenchymal stem cells, microenvironment

## Abstract

Lysosomal storage disorders (LSDs) constitute a group of monogenic systemic diseases resulting from deficiencies in specific lysosomal enzymes that cause the intralysosomal accumulation of non- or partially degraded substrates, leading to lysosomal dysfunction. In some cases of LSDs, the bone is more severely affected, thus producing skeletal manifestations in patients. Current therapies, such as enzyme replacement therapy (ERT) and gene therapy (GT), show limited efficacy in correcting skeletal abnormalities. Increasing evidence suggests that microenvironmental disturbances also contribute significantly to disease pathogenesis. Therefore, therapeutic strategies targeting lysosomal dysfunction and microenvironmental dysregulation are needed. Mesenchymal stem-cell-derived extracellular vesicles (MSC-EVs) are emerging as promising candidates in regenerative medicine due to their immunomodulatory, pro-regenerative, and paracrine properties. MSC-EVs have shown potential to modulate the microenvironment and favor tissue repair in bone-related disorders such as osteoarthritis and osteoporosis. Interestingly, MSC-EVs can be engineered to reach the bone and carry therapeutics, including ERT- and GT-related molecules, enabling targeted delivery to hard-to-reach bone regions. This review describes the main features of MSC-EVs and discusses the therapeutic potential of MSC-EVs as a potential cell-free strategy for bone-affected LSDs.

## 1. Introduction

Lysosomal storage disorders (LSDs) comprise a heterogeneous group of about 50 multisystemic genetic diseases caused by monogenic mutations in genes involved in lysosomal function [1,2]. Misfunctioned proteins lead to the lysosomal accumulation of partially or non-degraded substrates, resulting in a broad spectrum of clinical symptoms closely associated with the chemical nature of the accumulated compound [3].

Bone, one common tissue affected in several LSDs, comprises cells, minerals, and organic material [4,5]. Table 1 summarizes the LSDs affecting bone. The bone’s microstructure in adults involves a trabecular and cortical microarchitecture [6]. While the trabecular bone structure is significantly implicated in turnover, mineral homeostasis, and remodeling, the cortical bone is a low-pore and dense structure that mainly supports the mechanical strength of the bone [6,7].

Although several strategies have been tested for the treatment of bone-affected LSDs, including pharmacological chaperones (PCs) [8,9,10], enzyme replacement therapy (ERT) [11,12], substrate reduction/degradation therapy (SRT) [13,14], hematopoietic stem cell transplantation (HSCT) [15,16], and gene therapy (GT) [17,18], the bone pathology is, in most cases, poorly ameliorated. Although low drug penetrability into avascular zones of the bone seems to be the primary reason for the limited bone pathology correction [11,19,20], several further pathological mechanisms at the bone microenvironment, such as substrate accumulation-triggered chronic inflammation [21,22] and progenitor (i.e., mesenchymal stem cells, MSCs) and mature (i.e., chondrocytes, osteoblasts, osteoclasts, osteocytes) cells misfunction [22,23,24,25,26,27,28,29], could also play essential roles in bone manifestations.

**Table 1:** Common features of bone-affected LSDs.

LSD	Enzyme Deficiency	Accumulated Substrate	Symptoms	Ref.
GD I/III	GBA	GlcCer	osteopenia, sclerotic lesions, osteonecrosis, decrease mineralization.	[30]
ML II/III	GlcNAc-1-fosfo.	Mucolipids	dystosis multiplex, osteopenia, osteodystrophy, kyphosis, coarse facies.	[31]
MPS I	IDUA	DS/HS	dysostosis multiplex, kyphosis, coarse facies, short stature, hip dysplasia, pectus excavatum	[32]
MPS II	IDS	DS/HS	dysostosis multiplex, coarse facies, claw hands, kyphosis/gibbus, scoliosis, short stature, foot deformity.	[33]
MPS IIIA	SGSH	HS	Joint stiffness, contractures, dysostosis multiplex, scoliosis and hip dysplasia.	[34]
MPS IVA	GALNS	KS/C6S	dysostosis multiplex, pectus carinatum, gibbus, kyphosis, scoliosis, genu valgum, short stature, hypermobile joints, coarse facies	[35]
MPS VI	ARSB	DS	dysostosis multiplex, genu valgum, coarse facies, short stature	[36]
MPS VII	GUSB	HS, DS, and CS	dysostosis multiplex, coarse facies, joint contractures, genu valgum, short stature.	[37]
NPD-B	ASMase	Sphingo.	delayed skeletal maturation, osteopenia, osteoporosis.	[38]
Mann.	α-Mannosidase	MCO	dysostosis multiple, coarse facies.	[39,40]
Galacto.	Cathepsin A	Sial-Oligo	dysostosis multiple, coarse facial features.	[41]
Sial-II	Neuraminidase	Sial-Oligo	coarse facies, dysostosis multiplex, kyphoscoliosis.	[42]
Aspartyl.	AGA	GlcNAc-Asn	osteoporosis, hypermobile joints, delayed skeletal maturation.	[43,44]

**AGA.** Aspartylglucosaminidase. **ARSB.** Arylsulfatase B. **ASMase.** Acid sphingomyelinase. **Aspartyl.** Aspartylglucosaminuria. **Galacto.** Galactosialidosis. **GALNS.** N-acetylgalactosamine-6-sulfatase. **GBA.** Glucocerebrosidase. **GD.** Gaucher Disease. **GlcCer.** Glucocerebroside. **GlcNAc-1-fosfo.** GlcNAc-1-fosfotransferasa. **GlcNAc-Asn.** N-acetylglucosamine linked to asparagine. **GUSB.** β-glucuronidase. **Mann.** Mannosidosis. **MCO.** Mannose-containing oligosaccharides. **ML.** Mucolipidoses. **MPS.** Mucopolysaccharidosis. **GlcNAc-1-fosfotransferasa.** N-acetilglucosamina-1-fosfotransferasa. **IDUA.** α-l-iduronidase. **IDS.** Iduronato-2-sulfatasa. **NPD-B.** Niemann–Pick type B. **Sial.** Sialidosis. **Sial-Oligo.** Sialylated glycoproteins and oligosaccharides. **SGSH.** N-sulfoglucosamine sulfohydrolase. **Sphingo.** Sphingomyelin.

MSCs are well-recognized as potent immunomodulators by participating in both innate and adaptive immunity [45,46,47]. MSCs’ immunomodulatory capacity can be exerted via cell-to-cell interaction and paracrine activity [46]. Paracrine activity relies on the MSCs’ secretome, which comprises free soluble factors (i.e., cytokines, growth factors, and chemokines) and extracellular vesicles (EVs) [48,49]. EVs are cell-derived membranous structures with bioactive molecules that can modify local and distal cell fate [50,51,52,53]. EVs include exosomes, microvesicles, and apoptotic bodies [50]. Although MSC transplantation is being explored in several diseases using both autologous and allogenic transplantation as they are considered immuno-privileged [54], their low abundance (0.001–0.01% in bone marrow (BM)), potential immune reaction (for allogenic transplantation), donor–donor heterogeneity, fast clearance upon systemic infusion, and in vivo MSC misfunctioning rise as critical concerns [47,48,55]. Conversely, MSC-derived EVs (MSC-EVs) have a lower risk of immune reaction, can cross biological barriers, reach hard-to-treat tissues, and remain in the bloodstream for a prolonged period [52,56,57].

MSC-EVs garnered attention for their potential in treating bone diseases, including osteoarthritis [58,59], osteoporosis [60,61], and bone fractures [62,63], due to their immunomodulatory and regenerative properties. Most interestingly, MSCs can be engineered to enrich MSC-EVs with either enzyme or GT products, thereby acting as a dual system that transports and delivers therapeutics while immunomodulating the bone microenvironment [57,64,65]. Although in vivo data suggest that MSC-EVs conserve bone tropism upon systemic delivery [64,66], MSC-EVs can be further engineered to express membrane-anchored proteins able to interact with specific receptors on the plasma membrane of targeted cells [50,66,67], thus providing a promising cell-free approach to treat bone-affected LSDs.

This manuscript describes the principal features of MSC-EVs and discusses their potential as a cell-free alternative for bone-affected LSDs.

## 2. The Bone: Structure and Microenvironment

**Figure 1:**
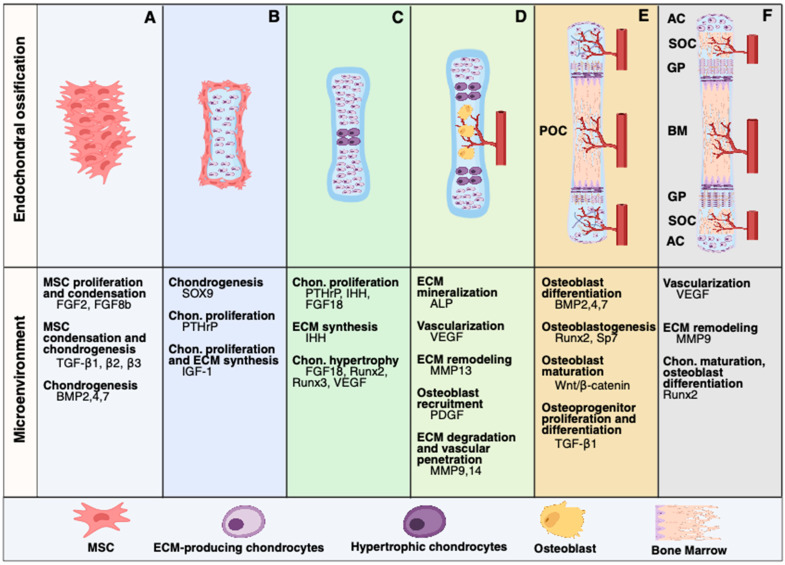
Endochondral ossification and microenvironment. (**A**) Mesenchymal stem cell (MSC) proliferation and condensation are driven by the transforming growth factor-beta (TGF-β1, 2, and 3) and fibroblast growth factors (FGFs2 and 8b) [76]. The bone morphogenetic proteins (BMP2, 4, and 7) contribute to MSC regulation while activating chondrogenesis via SOX9 expression in conjunction with TGF-β [77]. (**B**) Chondrogenesis is followed by chondrocyte proliferation maintained by parathyroid hormone-related protein (PTHrP). PTHrP also prevents premature chondrocyte hypertrophy. Insulin-like growth factor 1 (IGF-1) supports chondrocyte proliferation and the synthesis of extracellular matrix (ECM) [78]. (**C**) PTHrP continues to prevent chondrocyte hypertrophy through a feedback loop mediated by Indian hedgehog (IHH). FGF18, Runx 2, and 3 regulate chondrocyte hypertrophy. Hypertrophic chondrocytes secrete vascular endothelial growth factor (VEGF), promoting vascularization [78]. (**D**) Alkaline phosphatase (ALP) presence facilitates hydroxyapatite deposition [78]. VEGF facilitates further vascular invasion, while matrix metalloproteinase 13 (MMP13) enables cartilage resorption and remodeling [79]. Platelet-derived growth factor (PDGF) is involved in recruiting pericytes and osteoprogenitors. MMP9 and MMP14 contribute to cartilage degradation and vascular penetration [79]. (**E**) BMP2, 4, and 7 favor osteoblast differentiation [79]. Runx2 and Osterix (Sp7) activation are pivotal for osteoblastogenesis. Chondrogenesis is then suppressed by Wnt/β-catenin signaling, while osteoblast maturation is enhanced [80]. TGF-β1 stimulates osteoprogenitor proliferation, differentiation, and ECM turnover, forming primary ossification centers (POC) [80,81]. (**F**) VEGF leads to vascularization, enabling the formation of secondary ossification centers (SOC). ECM remodeling is mediated by MM9 activity, while Runx2 induces chondrocyte maturation and osteoblast differentiation [80,81]. This figure was created with Biorender.com.

Bone is a highly dynamic tissue composed of organic (e.g., collagen) and inorganic (e.g., hydroxyapatite) components, along with several cell lineages (e.g., osteoblasts, osteoclasts, osteocytes, chondrocytes) [68,69]. Bone originates from intramembranous or endochondral ossification from mesenchymal tissue [69,70,71]. While intramembranous ossification involves the direct osteoblast formation from MSCs, the endochondral ossification requires the differentiation of MSCs into chondrocytes to form hyaline cartilage, followed by the formation of primary and secondary ossification centers (Figure 1) [69]. Ossification involves the secretion of proteins and polysaccharides, which form a three-dimensional extracellular matrix (ECM) structure [72]. Whereas mechanical and biochemical properties precisely regulate bone ECM, the ECM actively participates in cell adhesion, proliferation, differentiation, and response to growth factors via signaling pathways [69,72,73]. Indeed, ECM regulates MSCs’ behavior and fate by providing spatially controlled growth factors to the MSCs, such as TGF-β, BMPs, FGF, and VEGF, as well as interaction of ECM proteins with MSCs integrins, including α5β1 and αvβ3, that promotes activation of several pathways involving FAK, MAPK, and PI3K/Akt, among others (Figure 1) [74,75].

In bone-affected LSDs, the skeletal manifestations are partially understood; nevertheless, substrate accumulation, inflammation, and complex interactions in the bone microenvironment could contribute to bone pathology [25]. Early studies conducted by Lecourt et al. (2013) in Gaucher disease (GD) showed that inhibition of GBA by Conduritol b epoxide (CBE) in healthy BM-derived MSCs led to a significant impairment in MSCs proliferation, which was attributed to G2/M cell cycle arrest [24]. Later studies confirmed the findings reported by Lecourt et al. and found an enhanced susceptibility to undergo apoptosis and senescence in MSCs from GD patients, due to impaired autophagy and DNA repair capacity [23]. Moreover, CBE-mediated GBA inhibition results in the overexpression of pro-inflammatory modulators, including MCP-1, IL-8, IL-6, DKK1, and SDF1 [23], which may contribute to increased bone resorption activity and osteoclast formation in GD. Later studies performed by Reed et al. (2018) also demonstrated slow proliferation of BM-derived MSCs from GD patients, along with a reduced osteoblast differentiation potential, low osteoblast-mediated calcium deposition, and increased osteoclast activity [26], further suggesting uncoupling between osteoblasts/osteoclasts that favors exacerbated osteoclastogenesis activation [27] in the GD bone microenvironment.

In MPS, the accumulation of GAGs is often found to disturb the normal development of chondrocytes at the growth plate [3]. Chondrocytes are MSC-derived cells that produce and maintain the ECM in the cartilage [82]. They are usually observed as enlarged and vacuole-filled cells, resulting in a disorganized columnar architecture in the proliferative and hypertrophic zone in MPS I [83], MPS IVA [84], MPS VI [85], and MPS VII [86]. Among several microenvironmental alterations, it has been postulated that the overexpression of SOX9 may contribute to a delay in chondrocyte hypertrophic differentiation [87], while the downregulation of STAT3 leads to reduced chondrocyte proliferation [28]. Similarly, chronic TLR-4-triggered pro-inflammatory states are also observed in MPS [21,22]. It is demonstrated that some GAGs, such as HS, can interact with TLR-4 and activate Myd88, which ultimately mediates the transcription of several inflammatory molecules, including IL-1β, IL-18, TNF-α, and matrix metalloproteinases (MMPs) [21,22]. MMPs are enzymes involved in ECM remodeling [88], and they are found to be increased in MPS IVA patients [29], potentially contributing to bone abnormalities by increasing ECM degradation in the bone microenvironment.

As many of the bone manifestations in LSDs are mediated by complex interactions in the bone microenvironment, attempting strategies able to modify that microenvironment could offer a novel approach for ameliorating bone symptoms. The upcoming sections will explore the use of native and engineered MSC-EVs as an innovative alternative for treating LSDs.

## 3. MSCs and MSC-EVs

MSCs are multipotent stem cells found in several tissues, including bone marrow (BM-MSCs), adipose tissue (AD-MSCs), skeletal muscle (SM-MSCs), placenta (P-MSCs), the dental pulp (DP-MSCs), and the umbilical cord (UC-MSCs) [89]. As multipotent cells, MSCs can differentiate into multiple lineages in response to specific signals, such as osteocytes, chondrocytes, as well as fat and muscle cells [54,89]. MSC transplantation was tested in some central and peripheral nervous system-affecting LSDs, including Krabbe [90], metachromatic leukodystrophy (MLD) [91], and Niemann–Pick type C [92].

In bone-affected LSDs, the evidence is minimal. Nonetheless, in bone diseases such as hypophosphatasia (HPP), allogenic MSC transplantation in two HPP patients who underwent BM transplantation led to increased bone mineralization [93]. Collectively, these data suggest that MSC transplantation alone or in combination with BM transplantation could exert therapeutic benefits in LSDs. Although the efficacy of MSC transplantation has been demonstrated, potential side effects remain a concern [94]. Thromboembolism [95] and capillary leak syndrome [96], among others, are complications associated with MSC transplantation [97], which can be mitigated by using the secretome of MSCs.

### 3.1. MSC Secretome

The MSC’s secretome is a unique source of several soluble molecules, primarily involving cytokines, chemokines, growth factors, and MSC-EVs [98]. Unlike MSCs, EVs are non-replicative, less immunogenic, more stable, and easier to handle. Moreover, EVs can be engineered to transport and deliver cargo to hard-to-reach tissues via ligand-receptor interactions [99]. MSC-EVs include exosomes, microvesicles, and large vesicles/apoptotic bodies [99]. Table 2 summarizes clinical trials using MSC-EVs for bone diseases [100].

#### 3.1.1. Exosomes

**Figure 2:**
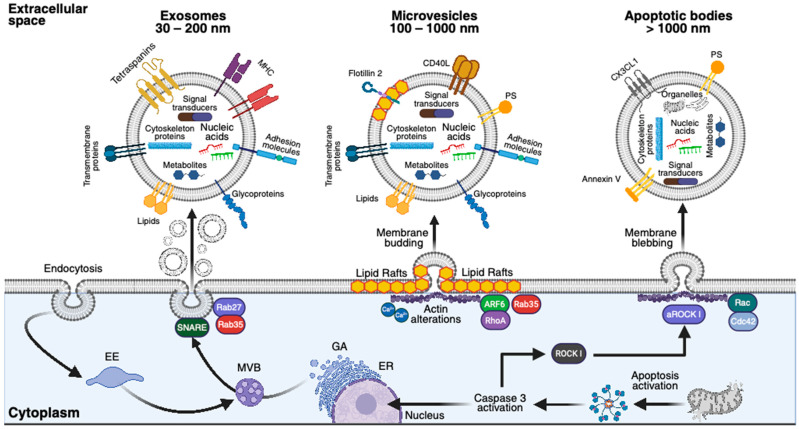
Extracellular vesicle biogenesis. Schematic representation of the three main classes of EVs—exosomes (30–200 nm), microvesicles (100–1000 nm), and apoptotic bodies (>1000 nm). Exosomes originate from the endosomal system via the maturation of early endosomes (EE) into multivesicular bodies (MVBs), followed by fusion with the plasma membrane. This process involves Rab GTPases (Rab27, Rab35) and SNARE proteins [99,104]. Classical surface markers include major histocompatibility complex (MHC) class I and II molecules, adhesion proteins (e.g., integrins), tetraspanins (e.g., CD9, CD63, and CD81), as well as biogenesis-associated proteins such as ESCRT components, ALIX, and TSG101 [105,106]. Microvesicles are formed by outward blebbing and subsequent fission of the plasma membrane in response to cytoskeletal contractility changes. They are highly dependent on membrane lipid composition, particularly raft-like domains. Shedding is calcium-dependent and regulated by ARF6, RhoA, and ROCK1 [106]. Common surface markers include CD40L, flotillin-2, and phosphatidylserine (PS). Apoptotic bodies are released through membrane blebbing during apoptosis and are characterized by the surface exposure of PS, Annexin V, and CX3CL1. Early steps involve caspase-3-mediated cleavage of Rho-associated kinase I (ROCK1). Activated ROCK1 (aROCK1) promotes actin-myosin contractility necessary for membrane blebbing [107]. Rac and Cdc42 are also implicated in cytoskeletal remodeling during this process. All EV types carry a broad spectrum of bioactive molecules, including lipids, cytoskeletal proteins, signal transducers, nucleic acids (DNA, mRNA, miRNA), and metabolites. Apoptotic bodies additionally contain intact cellular organelles. This figure was created with Biorender.com.

Initially identified as a cellular mechanism to mediate the exocytosis of unwanted cellular products, exosomes are endocytosis-originated EVs playing critical roles in cellular intercommunication by carrying and delivering functional proteins, metabolites, and nucleic acids (Figure 2) [99]. Typically, exosomes range in size from 30 to 200 nm and carry secondary metabolites, mRNA, miRNA, and other non-coding RNAs, as well as various proteins and lipids. Although specific molecular content depends on the cell source, about 80% of exosome proteins are highly conserved [101]. Exosomes are also characterized by expressing the tetraspanins CD9, CD63, and CD81, along with TS101, flotillin, Alix, and ESCORT3 (Figure 2) [102,103].

#### 3.1.2. Microvesicles

Microvesicles, or ectosomes, are medium-sized EVs ranging from 100 to 1000 nm that originate from plasma membrane budding in a calcium-dependent way. Like exosomes, microvesicles also carry proteins, lipids, metabolites, and nucleic acids. Microvesicles express integrins, selectins, CD40L, flotillin 2, and phosphatidylserine, which mediate intracellular communication (Figure 2) [99,104].

#### 3.1.3. Apoptotic Bodies

Apoptotic bodies constitute the largest EVs (>1000 nm), forming during late apoptosis through plasma membrane blebbing. Although apoptotic bodies can carry some biomolecules, such as those observed in exosomes and microvesicles, they also contain organelles. Annexin V, phosphatidylserine, and CX3CL1 are the most common surface markers (Figure 2) [99,104].

## 4. MSC-EVs and Bone-Affected LSDs

**Figure 3:**
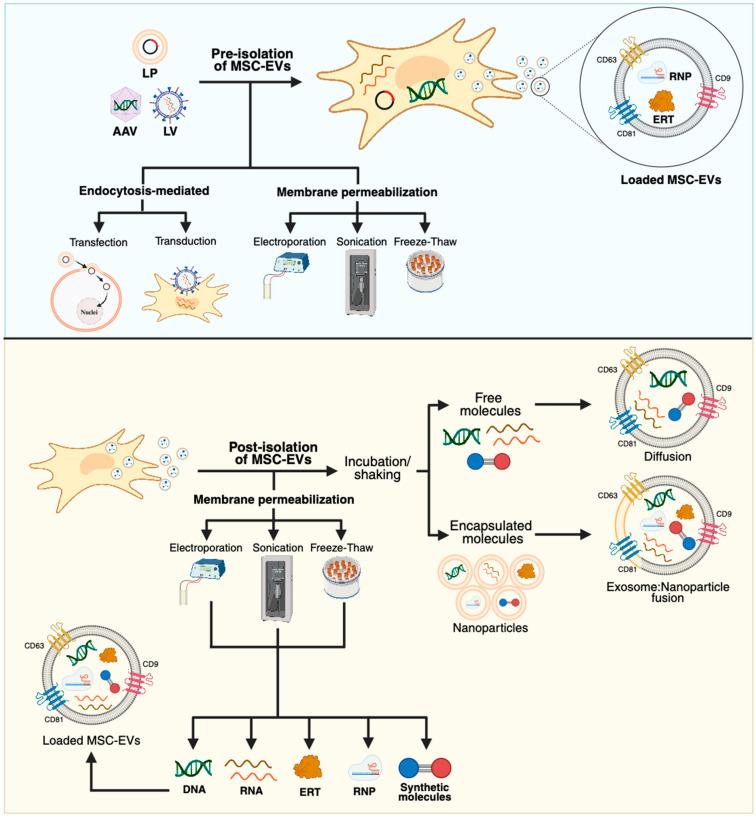
MSC-EVs loading strategies. (**Upper panel**) Genetic modification of MSCs using viral (VV) or non-viral vectors (NVVs) to induce the expression of a protein of interest enables the loading of MSC-EVs before their isolation [117,118]. Lipoplexes, along with other NVVs, typically mediate the transfer of genetic material through endocytosis-mediated delivery. VV, such as adeno-associated and lentiviral viruses, exploit their cell-invasive properties to enter the cell. Inducing membrane permeabilization via electroporation, sonication, or freeze-thaw cycles may increase VV, NVV, or internalization of free molecules within MSCs, then facilitating the incorporation of cargo (i.e., DNA, RNA, RNP, ribonucleoprotein (RNP) during EVs biogenesis [117]. (**Bottom panel**) EVs are also suitable for being loaded upon their release from MSCs. They can be loaded by simply incubating MSC-EVs in the presence of therapeutic molecules, such as nucleic acids and synthetic molecules, while shaking. Fusion with nanoparticles is also depicted as a strategy to encapsulate biomolecules into MSC-EVs. Membrane permeabilization is utilized to enhance loading efficiency [117,118]. The resulting loaded MSC-EVs are enriched with bioactive molecules, making them suitable for potential therapeutic applications. MSC-EVs loading does not alter classical surface markers (i.e., CD9, CD63, CD81), leading to their later purification using methods such as immuno-capture [117]. This figure was created with Biorender.com.

The natural ability of MSC-EVs to transport and deliver bioactive molecules can be used to design MSC-EVs that transport specific molecules [65,108]. While co-expressing proteins inside the MSCs can favor the cargo internalization within exosomes, loading exosomes upon their isolation is also feasible (Figure 3) [109]. Previous reports have shown that bioactive lysosomal enzymes can be released in EVs from various sources, including HEK293 [110], CHO [111], macrophages [112], BM-MSCs [113], and UC-MSCs [114].

### 4.1. MSC-EVs and ERT

MSC-EVs are naturally internalized by cells through endocytosis-dependent mechanisms, involving clathrin-mediated, caveolin-dependent, and macropinocytosis routes [115]. Upon uptake, EVs traffic from early endosomes through the endosomal pathway, ultimately reaching the lysosomes. This naturally occurring cell uptake significantly enhances the therapeutic delivery of recombinant enzymes directly into lysosomes, where they can exert their degradation activity on accumulated substrates [115], thereby ameliorating cellular stress.

Early studies conducted by Do et al. (2019) [116] aimed to express GBA fused to the exosome-anchoring vesicular stomatitis virus glycoprotein (VSVG) in HEK293 cells. GBA enzyme activity assays demonstrated that engineered EVs showed a 4.2-fold increase compared to native EVs [116], suggesting that implementing anchoring proteins such as VSVG could enhance the loading of lysosomal enzymes into EVs. Incubation of GBA-overexpressing EVs with HEK293 cells resulted in significant GBA intracellular activity and lysosome sorting [116], thus providing evidence for EVs enrichment with lysosomal enzymes involved in bone-affected LSDs.

Most recently, Flanagan et al. (2021) [114] showed that naturally occurring UC-MSC-EVs carrying the GALNS enzyme can be further engineered to overexpress GALNS via stable UC-MSC transfection. Co-culture of MPS IVA fibroblasts with GALNS-carrying UC-MSC-EVs resulted in a mannose 6-phosphate (M6P)-independent uptake by MPS IVA fibroblasts [114]. The MSC-EVs uptake mechanism could be mediated by global endocytosis routes involving clathrin/caveolin [119] rather than the classical interaction of M6P and the M6P receptor observed for free lysosomal enzymes [3]. Although phenotype correction was not extensively evaluated [114], these proof-of-concept experiments highlight the potential of MSC-EVs as a novel ERT approach for treating MPS IVA. In vivo experiments are still required to assess the therapeutic effect of GALNS-carrying UC-MSC-EVs in MPS IVA mice.

### 4.2. MSC-EVs and GT

Although EVs have been explored as a potential ERT approach for transporting and delivering several lysosomal enzymes [110,111,112,113,114], they can also be modified to transport GT vectors, such as the CRISPR/Cas9 system. The CRISPR/Cas9 system is a revolutionary genome editing tool that enables the rewriting of the genome by introducing expression cassettes at specific genomic regions and directly correcting point mutations [120,121]. The CRISPR/Cas9 system can be delivered as DNA, mRNA, or a preformed ribonucleoprotein (RNP) complex [120]. The CRISPR/Cas9 system can be loaded into EVs by either direct cell transfection or loading within isolated EVs (Figure 3) [122]. A comprehensive review discussing the advantages and limitations of each loading alternative was published by Berggreen et al. (2023) [122]. Notably, while EV-based ERT is intended to be sorted into the endosomal pathway to deliver the lysosomal enzyme within lysosomes, enabling native EVs to deliver the CRISPR/Cas9 system requires endosomal escape. The use of proteins, such as VSVG [123], listeriolysin O [124], and phospholipase C [125], has demonstrated endosomal escape properties and should be incorporated when engineering EVs to reduce their trafficking to lysosomes. The presence of viral or bacterial proteins raises immunological concerns, as they can activate immune responses [126]; therefore, their pro-inflammatory properties should be carefully assessed, particularly when attempting to treat bone-affected LSDs, in which inflammation at the bone microenvironment plays a key pathogenic role. Although no studies are testing the MSC-EVs-based CRISPR/Cas9 system delivery in bone-affected LSD models, growing evidence has demonstrated the efficacy of the CRISPR/Cas9 in ameliorating bone manifestations in some LSDs [18,127]. The use of MSC-EVs for transporting and delivering the CRISPR/Cas9 system could exert dual effects in correcting the mutation causing the LSDs while promoting microenvironment regulation.

## 5. MSC-EVs: A Perspective from Bone-Affected Non-LSDs

Only a few studies have tested the use of MSC-EVs for the specific treatment of bone-affected LSDs; nevertheless, several non-LSD models have demonstrated the feasibility of MSC-EVs in treating bone pathology by modulating the bone microenvironment via native and enhanced MSC-EV properties. This last section provides an overview of MSC-EVs for bone-involving diseases that could be implemented in bone-affected LSDs.

### 5.1. MSC-EVs’ Engineering—Therapeutic Molecules

In a study by Huang et al. (2023) [128], the feasibility of MSC-EVs engineered to overexpress miR-424 was tested in a rat calvarial bone defect model. In this study, the authors transduced MSCs with lentiviral particles containing a vector encoding miR-424 [128]. miR-424 upregulates expression of the bone morphogenic protein 2 (BMP-2) by negatively regulating SMAD7 and SMURF1 [129]. Initially, connective tissue removal was performed on the calvarial bone of anesthetized rats to create the calvarial defect, followed by local administration of a collagen plug containing engineered MSC-EVs [128]. Importantly, the authors evaluated the in vivo preservation of the immunomodulatory and reparative properties of the MSC-EVs by assessing the expression of induced nitric oxide synthase (iNOS) and Arg1, which serve as markers of pro-inflammatory and reparative properties, respectively [128]. MSC-EVs treatment induced a significant decrease in iNOS and an increase in Arg1 in calvaria wounds compared to untreated rats, supporting the notion that engineering MSC-EVs for overexpressing miR-424 did not alter the properties of MSC-EVs. Moreover, evaluation of bone regeneration carried out through micro-CT measurement revealed increased bone formation in miR-424-overexpressing MSC-EVs-treated rats compared to untreated or non-miR-424-overexpressing MSC-EVs, which was associated with a modest increase in the BMP-2 staining [128].

Although it requires further exploration, in MPS, it has been suggested that the delay in forming primary and secondary ossification centers is due to altered hypertrophic differentiation [130]. This appears to be mediated by impaired osteogenic signaling involving the Wnt/β-catenin and BMP pathways [130,131], which could be modulated by engineered MSC-EVs expressing specific miRNAs. Similarly, a previous study by Rintz et al. (2023) showed that AAV8-mediated C-type natriuretic peptide (CNP) expression induces bone growth in MPS IVA mice, which correlates with increased chondrocyte proliferation, along with a more columnar chondrocyte organization and reduced chondrocyte size compared to that observed in untreated MPS IVA mice [132]. Surprisingly, a significant decrease in KS levels in plasma and bone was also observed upon CNP-AAV8 treatment, although GALNS was not concomitantly expressed or administered in MPS IVA mice [132]. These novel achievements support the idea that modulating molecular pathways involved in bone biogenesis and its remodeling could be a next-generation therapy to ameliorate bone manifestations in LSDs. The transport and delivery of pivotal molecular regulators could benefit from engineered MSC-EVs, as MSC-EVs retain their immunomodulatory properties upon engineering [128].

### 5.2. MSC-EVs’ Engineering—Targeting Bone/Cartilage

Even though intravenous administration of MSC-EVs primarily results in trapping in the liver, lung, and spleen, some BM-MSC-EVs can also reach the bone marrow [64], suggesting bone tropism [133]. The tropism of MSC-EVs highly depends on the cell source [134]. To increase bone tropism, the surface of MSC-EVs can be engineered to express specific bone-targeting peptides [69]. For instance, bone-targeting exosomes were designed by Cui et al. (2022) to deliver siRNA targeting Shn3 in an osteoporosis mouse model [135]. The Shn3 gene inhibits osteogenic differentiation and promotes osteoclast activity, thereby increasing bone resorption [136]. Bone-targeting exosomes were formed by combining exosomes with the peptide SDSSD (Ser, Asp, Ser, Ser, Asp), followed by loading exosomes with siShn3 via electroporation [135]. Upon intravenous administration, the authors observed a more substantial accumulation of SDSSD-MSC-EVs in the bone compared to MSC-EVs, suggesting that bone-targeting MSC-EVs could increase their accumulation within the bone while preserving some biodistribution in other organs [135]. Similar bone tropism has been observed in MSC-EVs functionalized with aptamers [137], alendronate [138], and dextran sulfate [139]. The preservation of both bone- and non-bone tissue biodistribution is particularly interesting for bone-affected LSDs, as LSDs also affect other tissues [1]. A comprehensive review discussing the biodistribution of EVs in non-bone tissues was recently published by Deng et al. (2024) [140].

Thus, accumulation in tissues such as the liver could lead to the permanent expression of the lysosomal enzyme when attempting GTs such as those based on the CRISPR/Cas9 system.

Regarding chondrocytes, in vitro studies have suggested that chondrocytes take up MSC-EVs, leading to the upregulation of type II collagen expression and promoting ECM production and chondrogenesis [141,142]. Collectively, these studies support the idea that MSC-EVs exert cartilage regeneration. On the other hand, the microenvironment in the bone/cartilage is believed to exert pathogenic signaling, exacerbating the pathophysiology of LSDs. Using synovium and cartilage explants exposed to TNFα and IFNγ, early studies conducted by van Buul et al. (2012) showed that MSC-EVs shift the pro-inflammatory microenvironment towards an anti-inflammatory profile characterized by decreased expression of IL-1β, MMP-1, and MMP-13, as well as nitric oxide [143], suggesting that MSC-EVs promote immune regulation and ECM turnover. Most recently, Scalzone et al. (2024) [144] showed that the pro-inflammatory profile observed in human osteoarthritis (OA)-derived chondrocytes (OAC) can be regulated by MSC-EV treatment. OAC incubated with MSC-EVs decreases IL-8 and IFN-γ production while increasing IL-13. Likewise, MSC-EV treatment also led to a significant decrease in MMP13, which seems to contribute to cartilage abnormalities in LSDs [25]. MSC-EV treatment additionally restores ATP production in OAC [144]. We recently reported that ATP production is decreased in MPS IVA chondrocytes as a consequence of impaired mitophagy [145]; thus, MSC-EVs could provide an alternative for ameliorating the bioenergetic homeostasis in bone-affected LSDs, such as MPS IVA. Indeed, several studies support that MSC-EVs could exert therapeutic benefits by transporting and delivering functional mitochondria [146,147], thereby recovering the mitochondrial dysfunction observed in some bone-affected LSDs [145,148].

Some in vivo studies in non-LSD in vivo models have also demonstrated the suitability of MSC-EVs for ameliorating chondrocyte dysfunction [149]. For instance, Chen et al. (2022) reported that the administration of intraarticular UC-MSCs into OA rats led to a decrease in the expression of MMP-13 and ADAMTS-5 in chondrocytes, while increasing type II collagen expression [150]. Most promisingly, the cartilage damage observed in untreated OA rats, characterized by rough and denuded cartilage surfaces, thin cartilage layers, and an increased calcified cartilage zone, was significantly improved upon UC-MSCs treatment, strongly supporting the notion that UC-MSCs effectively modulate the cartilage microenvironment alongside chondrocyte physiology [150].

## 6. Future Perspectives

The therapeutic panorama for skeletal manifestations in LSDs remains limited. Current treatments, including ERT and GT, often fail to correct pathological findings within avascular skeletal zones such as cartilage and the growth plate. In this context, MSC-EVs have emerged as a promising next-generation approach, offering several biological advantages, including low immunogenicity, inherent tissue tropism, potential engineering, and delivery of complex molecular cargo. Despite their therapeutic promise and the compelling evidence of efficacy in preclinical studies, several drawbacks must be addressed before MSC-EVs can be fully integrated into clinical practice (Table 3). In 2023, a position paper highlighting the minimal information for studies of extracellular vesicles (MISEV2023) was published. Scientists interested in EVs should follow these guidelines, as they will be critical for successfully translating MSC-EVs-based therapies [103].

**Figure 4:**
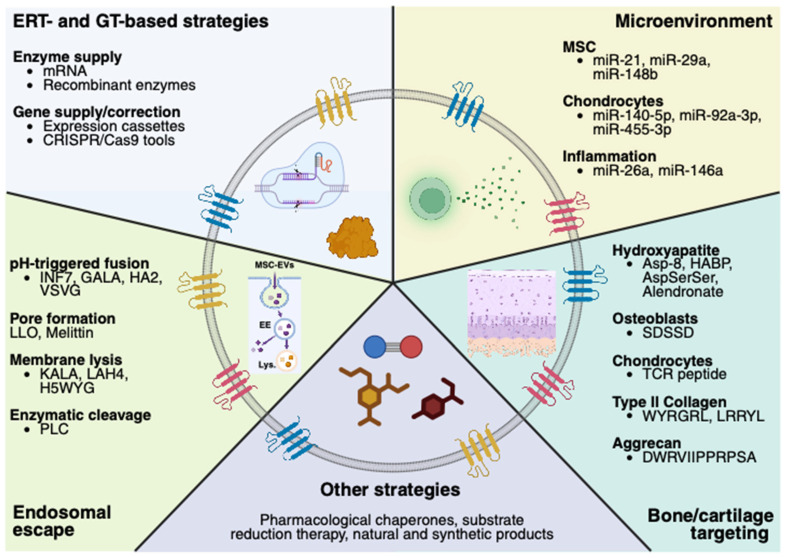
MSC-EVs as a potential alternative for bone-affected LSDs. Native and engineered MSC-EVs are emerging as alternatives for transporting and delivering therapeutics [66,114]. Endocytosis is the principal internalization pathway during EV uptake in mammalian cells. MSC-EVs’ endocytosis results in their trafficking from early endosomes (EE) to the lysosome (Lys.) [99], which results in successful ERT. Nevertheless, GT alternatives, such as the CRISPR/Cas9 system, require the vector to reach both the cytoplasm and the nucleus [159]; therefore, engineering MSC-EVs with endosomal escape molecules is critical in GT approaches [126]. Although it has not yet been tested in bone-affected LSDs, MSC-EVs could constitute a novel carrier of small molecules, as those used in pharmacological chaperones and substrate reduction therapy [1,13]. MSC-EVs are well-known for modulating the microenvironment [57,64,65] and could improve bone microenvironment alterations in LSDs [24,26,29,87] through miRNA overexpression [66,128,160,161,162]. A significant limitation of conventional therapeutics is their limited bioavailability within the avascular regions of the bone [19,163,164]. Functionalizing MSC-EVs with bone- or cartilage-targeting peptides may enhance their accumulation in skeletal tissues [69,137,138,165,166], improving therapeutic efficacy in bone-affected LSDs. This figure was created with Biorender.com.

MSC-EVs exhibit a multifaceted therapeutic profile. They can modulate the microenvironment by attenuating pro-inflammatory signaling, promoting tissue regeneration, and potentially delivering functional lysosomal enzymes or GT tools to anatomically inaccessible regions. Their biogenesis, cargo composition, and uptake mechanisms mirror those of native intercellular communication systems, making them particularly amenable to both systemic and local administration in LSDs. Advances in EV bioengineering further enable the functionalization of MSC-EVs’ surfaces to enhance targeting specificity. For example, conjugation with bone-targeting ligands, such as bisphosphonates, integrin-binding peptides, or aptamers, may facilitate selective homing to affected skeletal sites following intravenous or intra-articular delivery (Figure 4).

While EVs, particularly exosomes, hold promising potential in transporting and delivering specific cargos, their ability to avoid the immune response is also a significant advantage. For instance, a study conducted by Fu et al. (2021) showed that oncogene-targeting siRNA-carrying exosomes not only effectively decrease tumor formation in several cancer models but, most interestingly, their intravenous administration did not alter the profile of peripheral immune cells or pro-inflammatory cytokines [167], supporting the notion that exosomes are non-immunogenic platforms. As most of the bone-affected LSDs are also characterized by pro-inflammatory systemic profiles, the ability of native and engineered EVs to avoid triggering immune responses may offer a further advantage by preventing the exacerbation of the inflammatory environment in LSD patients.

On the other hand, the intracellular trafficking of MSC-EVs to lysosomes requires careful consideration. While lysosomal sorting is advantageous for ERT applications, as it leads to lysosomal enzyme delivery within the lysosome, it may limit the efficacy of GT cargos that require cytosolic or nuclear delivery. GT via MSC-EVs requires not only successful cellular uptake but also endosomal escape to allow gene-editing tools, such as CRISPR/Cas9, to access the cytoplasm and later the nucleus. Several bioengineering strategies have been proposed to facilitate this process, including the incorporation of viral proteins (i.e., VSVG), bacterial peptides (i.e., listeriolysin O), and membrane-disrupting enzymes (i.e., phospholipase C), as they induce membrane endosomal destabilization (Figure 4). Nevertheless, the use of these endosomal escape molecules raises several concerns. For instance, the use of viral and bacterial peptides can activate TLR-mediated responses [168], therefore exacerbating the preexisting inflammation profile, often observed in LSDs [3]. Likewise, an enhanced internalization of Cas9 proteins, when attempting CRISPR/Cas strategies, may lead to the persistence of Cas9 within cells, not only inducing immune responses against Cas9 but also increasing the potential for Cas9 off-targeting [169,170].

Finally, a further concern lies in the lack of quantitative data on EV biodistribution, cargo loading efficiency, and intracellular delivery routes in vivo. Therefore, the implementation of advanced imaging technologies, such as flow cytometry and single-particle tracking, may help to understand EV behavior more precisely, thereby accelerating the rational use of these promising MSC-EVs in clinical practice.

## Figures and Tables

**Table 2:** Clinical trials involving MSC-EVs for bone-affected diseases.

Trial ID	Status	Dis.	Source	Phase	DM	Dose Par/Dose	Outcomes Measured	Main Findings
NCT06713902	Recruiting	OA/KJ	AD	Obs.	NA	NA	Encapsulation of AD-MSCs into PRP-derived fibrin gel	NP
NCT05060107	Completed	OA/KJ	NA	I	IA	Single 3–5 × 10^11^	Safety, pain, and disability reduction	MSC-EVs were safe and reduced pain while improving function
NCT06431152	Recruiting	OA/KJ	UC	I	IA	Single L: 2 × 10^9^ M: 6 × 10^9^ H: 2 × 10^10^	Safety, pain, and disability reduction	NP
NCT06466850	Recruiting	OA/KJ	NA	I	IA	* Double	Safety, pain, and disability reduction	NP
NCT06463132	Not yet recruiting	OA/KJ	PL	I	IA	** Single	Safety, clinical improvements after 12 months	NP
NCT06713902	Recruiting	OA/KJ	AD	Obs.	NA	NA	Encapsulation of AD-MSCs into PRP-derived fibrin gel	NP
NCT06688318	Active, not recruiting	OA/KJ	UC	I/II	IA	** Single	Safety, pain reduction.	NP
NCT04998058	Not yet recruiting	EM	AD	I/II	MSL	** Single	Bone density and quantity	NP
NCT05261360	Unknown	DMI	SF	II	IA	*** 1 × 10^6^	Safety, pain reduction, cytokine profile	NP

* Intra-articular injection at day 1 and 90. Particle/dose is not disclosed. ** Particle/dose is not disclosed. *** This is referred to as MSCs/Kg exosomes. **AD.** Adipose. **Dis.** Disease. **DM.** Delivery method. **DMI.** Degenerative Meniscal Injury. **EM.** Edentulous maxilla. **H.** High. **IA.** Intra-articular. **L.** Low. **M.** Medium. **MSL.** Maxillary sinus lift bone grafting. **NA.** No available. **NP.** Not published yet. **OA/KJ.** Osteoarthritis of the knee joint. **Obs**. An observational study in which AD-MSCs are isolated from individuals undergoing plastic surgery procedures. **Par/dose.** Particles/dose. **PL.** Platelet. PRP. Platelet-rich plasma. **SF.** Synovial fluid. **UC.** Umbilical cord.

**Table 3:** Major drawbacks in MSC-EVs.

Drawback	Description	Ref.
Cargo heterogeneity	Cargo is greatly influenced by MSCs’ source, passage, and culture conditions.	[103,151]
Limited cargo loading	Low loading efficiency is often observed, mainly when passive (incubation/shaking) methods are used.	[152]
Non-standardized isolation protocols	Ultracentrifugation, density gradient centrifugation, size-exclusion chromatography, ultrafiltration, precipitation, and immunocapture yield different purities. Characterization procedures upon isolation are inconsistent, thereby limiting biochemical composition identification.	[153,154]
Leak of biodistribution assays	Although some studies use fluorescence-based MSC-EVs, this is not the case for all studies. It is also unclear how MSC-EVs are cleared.	[155,156]
Potential immunogenicity	Engineered MSC-EVs can increase the risk of immune response activation, thus exacerbating disease pathology.	[57,157]
Scalability	Large-scale production is still limited.	[158]

## Data Availability

Not applicable.

## Abbreviations

The following abbreviations are used in this manuscript:

AD-MSC	Adipose-derived mesenchymal stem cells
BM	Bone marrow
BM-MSCs	Bone marrow-derived mesenchymal stem cells
DP-MSCs	Dental pup-derived mesenchymal stem cells
ERT	Enzyme replacement therapy
GT	Gene therapy
MSCs	Mesenchymal stem cells
P-MSCs	Placenta-derived mesenchymal stem cells
SM-MSCs	Skeletal muscle-derived mesenchymal stem cells
UC-MSCs	Umbilical cord-derived mesenchymal stem cells
